# The Dynamics of miR-449a/c Expression during Uterine Cycles Are Associated with Endometrial Development

**DOI:** 10.3390/biology12010055

**Published:** 2022-12-29

**Authors:** Mladen Naydenov, Maria Nikolova, Apostol Apostolov, Ilias Glogovitis, Andres Salumets, Vesselin Baev, Galina Yahubyan

**Affiliations:** 1 Faculty of Biology, University of Plovdiv, Tzar Assen 24, 4000 Plovdiv, Bulgaria; 2 Center for Women’s Health, 4000 Plovdiv, Bulgaria; 3 Competence Centre on Health Technologies, 50406 Tartu, Estonia; 4 Department of Neurosurgery, Cancer Center Amsterdam, Amsterdam University Medical Centers, VU University Medical Center, De Boelelaan 1117, 1081 HV Amsterdam, The Netherlands; 5 Division of Obstetrics and Gynaecology, Department of Clinical Science, Intervention and Technology (CLINTEC), Karolinska Institute, Karolinska University Hospital, 14186 Stockholm, Sweden; 6 Department of Obstetrics and Gynecology, Institute of Clinical Medicine, University of Tartu, 50406 Tartu, Estonia

**Keywords:** microRNAs, isomiRs, miR-449 family, endometrial receptivity

## Abstract

**Simple Summary:**

The human endometrium is a highly dynamic tissue. Increasing evidence has shown that microRNAs play essential roles in human endometrium development during the menstrual cycle. Here, we applied small RNA-sequencing to demonstrate that miR-449a/c and their sequence variants (isomiRs) may participate in the genetic control of human endometrial receptivity. Stem-looped RT-qPCR analysis of miR expression at four time-points of the endometrial cycle verified the increased expression of the miR-449a/c family members in receptive endometrium, among which the 5′ isoform of miR-449c–miR-449c.1 was the most strongly up-regulated. Moreover, we found in a case study that the expression of miR-449c.1 and its precursor (pre-miR-449c) correlated with the histological assessment of the endometrial phase and patient age. We believe this study will promote the clinical investigation and application of the miR-449 family in the diagnosis and prognosis of human reproductive diseases.

**Abstract:**

The human endometrium is a highly dynamic tissue. Increasing evidence has shown that microRNAs (miRs) play essential roles in human endometrium development. Our previous assay, based on small RNA-sequencing (sRNA-seq) indicated the complexity and dynamics of numerous sequence variants of miRs (isomiRs) that can act together to control genes of functional relevance to the receptive endometrium (RE). Here, we used a greater average depth of sRNA-seq to detect poorly expressed small RNAs. The sequencing data confirmed the up-regulation of miR-449c and uncovered other members of the miR-449 family up-regulated in RE—among them miR-449a, as well as several isoforms of both miR-449a and miR-449c, while the third family member, miR-449b, was not identified. Stem-looped RT-qPCR analysis of miR expression at four-time points of the endometrial cycle verified the increased expression of the miR-449a/c family members in RE, among which the 5′ isoform of miR-449c–miR-449c.1 was the most strongly up-regulated. Moreover, we found in a case study that the expression of miR-449c.1 and its precursor correlated with the histological assessment of the endometrial phase and patient age. We believe this study will promote the clinical investigation and application of the miR-449 family in the diagnosis and prognosis of human reproductive diseases.

## 1. Introduction

The human endometrium plays a key role in establishing pregnancy. Communication between the lining of the uterus and the embryo is still largely enigmatic, despite the growing data gathered thanks to the tremendous advances in omics technology. It is well known that the endometrium is receptive during a short period of the menstrual cycle (the window of implantation, WOI), and successful implantation depends on the synchronization between the receptive endometrium (RE) and the quality blastocyst. Poor endometrial receptivity is thought to be a more common cause of recurrent implantation failure (RIF) than embryo quality [1,2]. In-depth knowledge of the molecules that control gene expression underlying endometrial receptivity opens new perspectives for understanding its nature at the molecular level and for rational interventions [3,4].

MicroRNAs (miRs), the most abundant class of small RNAs in animals, are encoded by MIR genes or other types of genomic loci [5,6,7], the transcription of which results in primary transcripts (pri-miRs) [8]. The latter form stem-loop structures and are sequentially cleaved by the RNase III enzymes Drosha and Dicer, to produce precursor miRs (pre-miRs) and mature miRs [9,10]. Processing can often generate multiple miR sequence variants—isomiRs. Cleavage by Drosha and/or Dicer is not always precise, and may also be affected by various internal cues leading to the formation of template isomiRs that fully match the precursor but have shifted 5′- and/or 3′-ends, compared to the reference miRs [11,12,13]. Furthermore, non-template isomiRs can arise from non-templated nucleotide additions (NTAs), mainly involving adenylation or uridylation at the miR 3′-ends [14]. MiRs are critical regulators of development, cell differentiation, and homeostasis [15].

The expression profiles of endometrial miRs in both regular and stimulated cycles have been studied using high throughput techniques, including microRNA microarrays [16,17] and sRNA-seq [18,19,20]. An increasing number of endometrial investigations focus on miRs to elucidate in depth the molecular origins of recurrent implantation failure (RIF). It has been shown that patients with infertility and those who have experienced RIF have disordered miR signatures [21,22]. Moreover, they have potential as diagnostic and prognostic biomarkers and as therapeutic molecules [23]. The complete analysis of cytologically different cells in the endometrium in the context of miR expression [24], together with exosomal miRs [25], aims to give additional and more accurate assessment for a better understanding of embryo–uterine crosstalk.

The miR-34/449 family comprises six homologous miRNAs (miR-34a, miR-34b, miR-34c, miR-449a, miR449b and miR-449c), which are encoded by three distinct loci (miR-34a, miR-34b/c and miR-449). MiR-34a is encoded separately on chromosome 1p36, and the miR-34b/c cluster is located on chromosome 11q23. The miR-449 cluster consists of three members, miR-449a, miR-449b, and miR-449c, and is located in the second intron of the Cdc20b gene on chromosome 5q11. Recent advances in next-generation-sequencing (NGS) technologies have made it possible to detect many miR sequence variants, or isomiRs [26,27,28]. There is increasing evidence suggesting that the miR-34/449 family comprises a variety of isomiRs. The identified 5′-isomiRs of miR-34b and miR-449c were more abundant than the reference miRs in the breast cancer cell line [29], human airway epithelial cell (HAEC) primary cultures [30], and endometrial tissue [31]. Moreover, these isomiRs share the same “seed” region with the other reference members of the miR-34/449 family. Thus, two subpopulations of sequences differing at the 5′-end and, therefore, at the “seed” region can be defined in the miR34/449 family, leading to an expansion of the family’s target genes. Several studies have shown that different family members may display functional variations or perform similar functions in different cellular contexts [30,32].

The miR-34/449 family members are potent inducers of cell-cycle exit, promoting epithelial cell differentiation [33,34,35,36]. They act as master regulators of motile ciliogenesis in vertebrates, and have been found highly expressed in human tissues containing large amounts of cilia, such as the airways, brain, and female and male reproductive tracts [37,38,39]; miR-34c is involved in normal spermatogenesis in mammals, and plays a pivotal role in sperm output [40,41,42]. Family members play a crucial role in inhibiting epithelial to mesenchymal transition (EMT), cancer-stem-cell formation, cancer invasion, and metastasis, thus contributing to tumor suppression [43,44].

In a previous study, we applied small RNA sequencing (sRNA-seq) to describe the dynamics of miR/isomiR expression in natural cycles primed by chorionic gonadotropin (hCG). Our data demonstrated the complexity and dynamics of endometrial isomiRs, which can act in concert with miRs to control functionally important genes critical for RE [31]. Here, we use a greater average depth of sRNA-seq to allow better coverage and the detection of poorly expressed small RNAs, and reveal the miR449 family, represented by two of the RefSeq miRs and five isomiRs, as the most highly regulated miR family in the RE of hCG-primed endometrial cycles. The expression profiles of miR449a/c were examined during the successive phases of the endometrial cycle using RT-qPCR. In a case study, miR-449c expression was correlated with endometrial histological pattern and patient age.

## 2. Materials and Methods

### 2.1. Patients, Study Design, and Samples

The Research Ethics Committee of the Faculty of Biology at Paisii Hilendarski University, Plovdiv, Bulgaria, gave its approval for the project. Every participant was a volunteer and gave written informed consent.

Participants in the study had to meet the following criteria: they all had regular menstrual cycles, a normal BMI, and no sexually transmitted infections, infertility-related diseases (hydrosalpinx, endometriosis, PCOS, fibroids, polyps, or any uterine abnormalities); nor did they smoke or use alcohol or drugs throughout the study period. Every woman had experienced a successful pregnancy and given birth to at least one child.

We performed folliculometry and endometrial-thickness measurements (Fukuda Denshi Full Digital Ultrasound System UF-870AG, Tokyo, Japan) starting on day 7, counted from the first day of menstrual bleeding. Blood tests for luteinizing hormone (LH), progestogen, and estradiol were performed from the beginning. Serial ultrasound measures and hormonal blood tests for estradiol and LH were carried out daily when a follicle measuring at least 17 mm was identified.

When the follicle was at least 18 mm, the endometrium was at least 6.5 mm, E2 was at least 130 pmol/L, and LH was less than 13 IU/l, ovulation was induced by the subcutaneous administration of Choriomon (hCG) 5000 UI (IBSA Farmaceutical Italia S.r.l, Lodi, Italy). Probette was used to conduct outpatient endometrial biopsies on each patient without anesthesia (endometrial microbiopsy curette).

The individual biopsies were collected on the day when the requirements for hCG administration were satisfied, as well as on days 2, 7, and 9 after the application of hCG, corresponding to the secretory phase. The biopsies were taken at four different time-points throughout the same menstrual cycle, and corresponded to the proliferative phase. Each biopsy had a piece removed and placed in 10% buffered formalin for later histological analysis. Thermo Fisher Scientific’s RNAlater was added to another portion, which was then supplemented and kept at −80 °C, until use.

### 2.2. Histology

The endometrial biopsy was subjected to classical histological examination by staining with hematoxylin-eosin (H-E), and morphological evaluation was carried out using Noyes criteria [45].

### 2.3. RNA Extraction and Quality Control

Total RNA was extracted and purified from 50 mg of endometrial tissue using NucleoSpin miRNA (Macherey-Nagel, Germany) in combination with QIAzol Lysis Reagent (Qiagen, Germany). The RNA quality and quantity were checked with a Qubit 4 Fluorometer (Invitrogen™, Thermo Fisher Scientific, Waltham, MA, USA). Samples with an RNA integrity number (RIN) ≥ 7 determined by the Qubit RNA IQ assay were used for further analysis.

### 2.4. sRNA-Seq and Data Analysis

The sRNA-seq of endometrial samples was performed according to published protocols [46], with 5 ng of total RNA as input. Purified RNA from each library was collected and sequenced using the Illumina NovaSeq 6000 platform at Single-end 50 bp, 10M reads per sample (Novogene Europe, UK). QC verification and adapter trimming of the FASTQ files were performed using FastQC and Trim Galore via the miRGalaxy platform [47] (https://hub.docker.com/r/glogobyte/mirgalaxy, accessed on 1 December 2022). The same platform was used to identify and classify miRs and template and non-template isomiRs, based on read mapping against reference (RefSeq) miRs and read copy-number. For differential expression analysis, the count matrices of miRs and isomiRs in each sample were forwarded to Limma software (Galaxy Version 3.38.3) with the options of log2FC > 1.5 and *p*-value adjusted threshold < 0.05.

### 2.5. miR, isomiR and Pre-miR Expression Profiling by RT-qPCR

Reverse transcription (RT) was carried out with the Revert Aid H Minus First Strand cDNA Synthesis kit (Thermo Fisher Scientific, Waltham, MA, USA). RT of miRs and isomiRs to cDNA was performed with stem-loop (SL) primers [48] or two-tailed (TT) primers [46], while random hexamers were used for the RT of pre-miRs. The conditions used for the RT of miRs and isomiRs were: 16 °C for 30 min, followed by 60 amplification cycles at 30 °C for 30 s, 42 °C for 30 s, 50 °C for 1 s, and a final step at 70 °C for 5 min. The synthesis of cDNA of pre-miR was carried out according to the manufacturer’s instructions.

The qPCR reactions were run on Applied Biosystems 7500, using TaqMan™ Universal Master Mix II, no UNG (Thermo Fisher Scientific, Waltham, MA, USA) for miRs and isomiRs and a Green Master mix kit for pre-miRs (Genaxxon Bioscience). The following conditions were used for each amplification product: 95 °C for 10 min, followed by 40 amplification cycles at 95 °C for 10 s, and 60 °C for 1 min. RNU48 was used as an endogenous control to normalize each gene expression level.

The designed SL primers, TT SL primers, TaqMan probes for miRs, isomiRs, and pre-miRs are listed in Table S1. For each RT-qPCR reaction, three technical replicates were performed. Relative quantification (RQ) was calculated using the 2−ΔΔCt method, relative to the endogenous control.

## 3. Results

### 3.1. sRNA-Seq Expression Data Identified the miR-449 Family as the Most Strongly Up-Regulated in the hCG-Primed RE

**Scheme 1:**
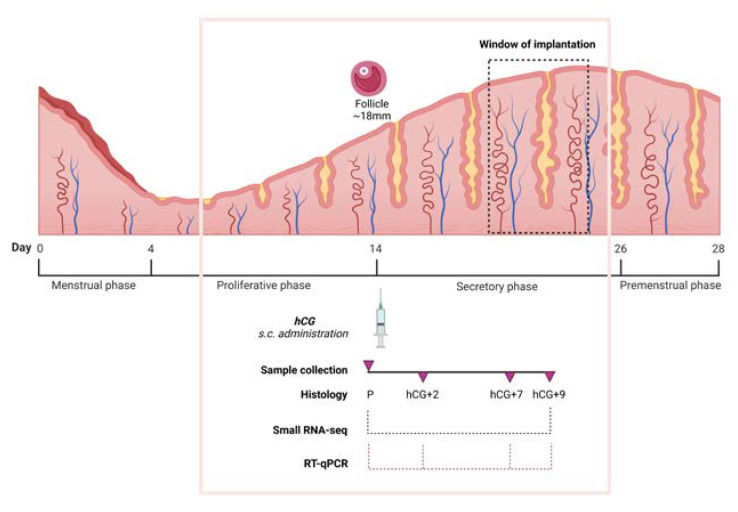
Experimental settings. Choriomon (hCG) was administered subcutaneously (s.c.) when the follicle was at least 18 mm, and the endometrium met the requirements as described in Materials and Methods. The first time-point for tissue collection was before hCG administration, and corresponded to the proliferative phase (P). Subsequent tissue collection time-points were 2, 7, and 9 days after hCG administration, hCG + 2, hCG + 7, and hCG + 9, respectively, and corresponded to the secretory phase. All tissue samples were collected from one menstrual cycle of the respective patient. P and hCG + 9 samples were analyzed using small RNA-seq. P, hCG + 2, hCG + 7, and hCG + 9 samples were analyzed using stem-loop RT-qPCR. Created with BioRender.com.

Administration of hCG is considered to cause ovulation within 36–48 h, and allows for more accurate secretory-phase dating. In our previous study, the leading indicator in the formation of the studied groups of women for sRNA-seq analysis was the day after the hCG priming—2, 7, or 9 days after hormone administration [31]. However, the histological description of some patients’ endometrium showed slight time shifts according to the Noyes criteria for the respective phases of the cycle, most likely due to inter-subject variability. For example, the oldest patient in the cohort analyzed in the previous sRNA-seq-based profiling showed a shortening of the secretory phase, compared with younger patients. In the present study, we selected six women in full compliance with the Noyes criteria for collecting endometrial samples that corresponded to the proliferative (P) phase and the mid-secretory (MS) phase (at hCG + 9), when the endometrium is most receptive to blastocyst implantation (Scheme 1). Here, sRNA-seq was performed at Novogene Bioinformatics Technology Co., Ltd. with an average sequencing depth of 10 M reads. To detect miR and isomiR candidates, the qualified reads were processed by the miRGalaxy pipeline [47].

**Figure 1:**
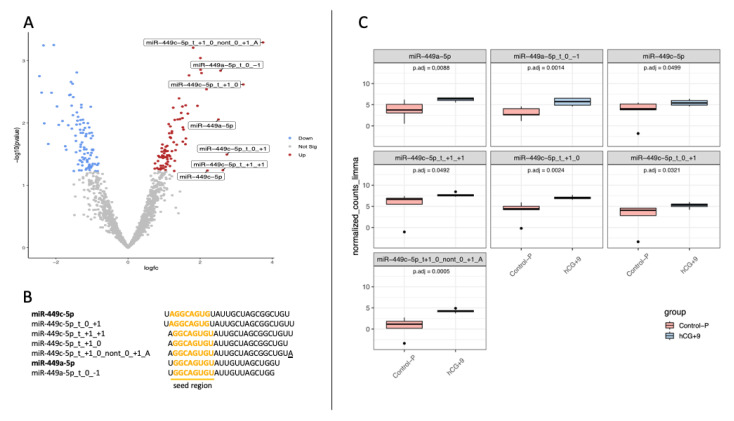
sRNA-seq expression data identified the miR-449 family as the most strongly up-regulated miR family in the hCG-primed receptive endometrium. (**A**) Volcano plot of DE miR and isomiRs in RE; (**B**) alignment of DE members of the family in RE with seed regions (indicated in yellow); (**C**) normalized expression of miRs and isomiRs of the family in proliferative phase (blue) and MS (hCG + 9) phase (yellow); (Limma, Galaxy Version 3.38.3). Adjusted *p*-values (p.adj) as generated by Limma, boxes span the 25th to 75th percentiles, whiskers extend to the 10th and 90th percentiles, and dots indicate outliers.

Analysis of sRNA-seq data identified 157 miRs and isomiRs with altered expression at the endometrial cycle’s P to MS phase transition (Table S2). Of these, the top seven most altered members belonged to only one family, miR-34/449 (Figure 1A). Compared to our previous study, where only two members of the miR-449 family showed expression changes in the MS phase [31], here we found a larger number of members upregulated in the MS phase. The family was represented by miR-449a-5p and one of its isomiR variants, miR-449c-5p, and four of its isomiR variants, while no representatives of miR-449b were found (Figure 1B). Two 5′offset isomiRs of miR-449c (miR-449c-5p_t_ + 1_0 and miR-449c-5p_t_ + 1_ + 1, where t stands for templated), were the most abundant family members (Figure 1C). MiR-449c-5p_t_ + 1_0 will be referred to hereafter as miR-449c.1. The only non-templated isomiR detected (miR-449c-5p_t_ + 1_0_nont_0_ + 1_A, where nont stands for non-templated), which is a variant of miR-449c.1, was the least abundant family member.

Of all DE miRs, miR-449 family members showed the highest differential expression values (log2FC, ranging from 2.20 to 3.75, *p* ≤ 0.05) in the MS endometrium, compared with P (Figure S1). Interestingly, isomiRs were more strongly up-regulated than their canonical counterparts. The most significant increase in relative expression was found for miR-449c.1 and its non-template variant.

### 3.2. Expression of the mir-449 Family Is Dynamic throughout the Endometrial Cycle, with a Peak in the Receptive Endometrium

**Figure 2:**
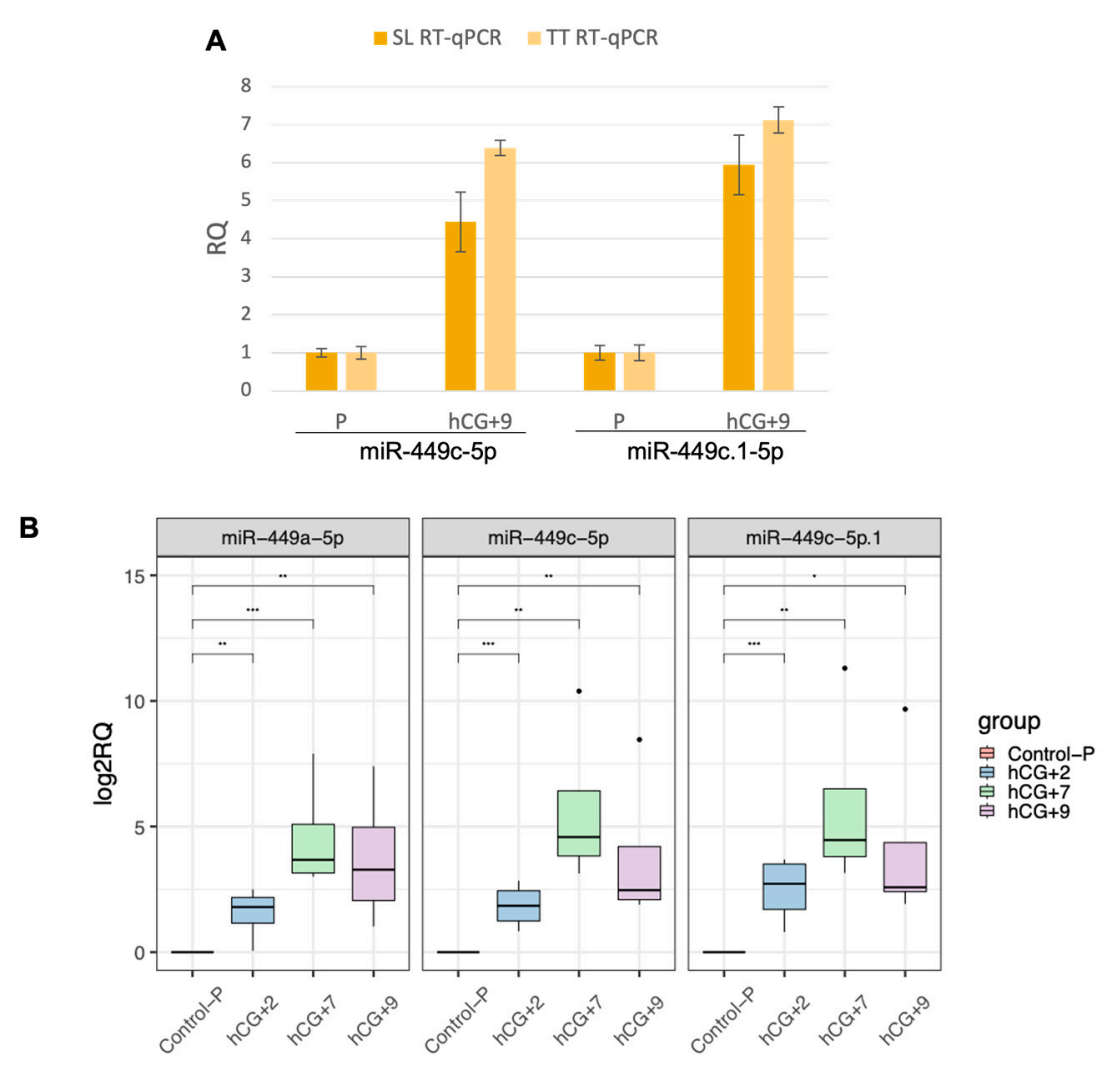
Expression of the mir-449 family throughout the endometrial cycle. (**A**) Relative quantity of miR-449c and miR-449c.1 analyzed using stem-loop (SL) RT-qPCR and two-tail (TT) RT-qPCR in one patient; error bars represent standard deviation of three technical repeats. (**B**) Relative expression levels of miR-449a, miR-449c, and miR-449c.1 analyzed using SL RT-qPCR in the patient cohort hCG + 2, hCG + 7 and hCG + 9 compared to proliferative phase (control-P). Expression changes among the groups were compared using one-way ANOVA and unpaired t-test (* *p* < 0.05; ** *p* < 0.01; *** *p* < 0.001); boxes span the 25th to 75th percentiles, whiskers extend to the 10th and 90th percentiles, and dots indicate outliers.

To verify the sRNA-seq expression data, we first examined the relative expression levels of miRs of interest by two target-specific RT-qPCR assays—SL RT-qPCR [48] and TT RT-qPCR [46] in one patient (three technical replicates). Two miR species were selected for this purpose—the canonical miR-449c and the most strongly up-regulated in the MS endometrium isoform—miR-449c.1, as indicated by the sRNA-seq data (Figure 2A). Both methods confirmed the increased expression levels of the two studied miR species in the MS phase, compared with the P phase. Based on the obtained expression profiles, it is difficult to assess to what extent each of the two methods distinguishes the two miR species, which differ by only 1 nt at the 5′-end, if possible. Therefore, we focused on the SL RT-qPCR for subsequent analyses.

The expression levels of the most abundant members—miR-449a, miR-449c, and miR-449c.1—were determined at four-time points: P, hCG + 2, hCG + 7, and hCG + 9, the last two of which fall into the MS phase. With the designed primers and probes for the three miR species (Table S1), we cannot exclude the possibility that the target miRNA is amplified together with some of its isoforms. The RT-qPCR results confirmed the increased expression of the miR-449a/c family members in the MS as determined by the sRNA-seq, with a peak appearing at hCG + 7, after which expression declined (Figure 2B).

### 3.3. miR-449c.1 Expression Correlates with Endometrial Histological Pattern and Patient Age (Case Study)

**Figure 3:**
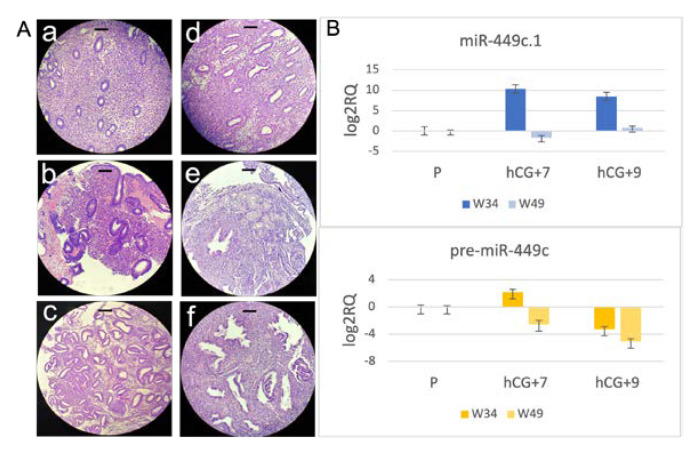
Correlation of miR-449c.1 and pre-miR449c expression with endometrial histological pattern in a case study. (**A**) Endometrial histological pattern of a 34-year-old woman (W34, (**a**–**c**)) and a 49-year-old woman (W49, (**d**–**f**)) at proliferative phase (**a**,**d**), hCG + 7 (**b**,**e**) and hCG + 9 (**c**,**f**), scale bars 50 μm. (**B**) Relative expression levels of miR-449c.1 (blue) and pre-miR449c (yellow) in W34 and W49 analyzed using SL RT-qPCR—hCG + 7 and hCG + 9 compared with the proliferative phase (P) of the corresponding woman. Error bars represent standard deviation of three technical repeats.

During the experimental procedure, we found a 49-year-old patient (W49) whose histological pattern differed from those of the six women selected for sRNA-seq analysis. She had regular menstruation but in a short period of cyclicity—an average of 25 days between cycles. What is seen on the histology is a shortening of the secretory phase with scattered irregular glands lined by columnar epithelium, sometimes with a back-to-back arrangement and stromal edema at the HCG + 9 point, which is more likely to be interpreted as a late secretory phase (Figure 3A).

We investigated the dynamics of the endometrial expression of miR-449c.1 in the W49 during the endometrial cycle. To understand whether the changes in miR-449 expression could be related to endometrial aging, we analyzed the expression dynamics in the younger woman (34-year-old, W34), the histological parameters of which were shifted in time according to the Noyes criteria for the corresponding phases of the endometrial cycle (Figure 3A). SL RT-qPCR was performed with the primers and probes designed for miR-449c.1. The expression profiles found were contrasting—the miR-449c.1. levels were up-regulated at MS in W34, while down-regulated in W49 (Figure 3B). To determine whether the observed contrast gene expression is a consequence of altered transcriptional control, we assessed the expression levels of the miR-449c precursor (pre-miR449c) using RT-qPCR in the endometrial samples of the two patients. The precursor expression-profile showed up-regulation in patient W34 and down-regulation in patient W49 at the hCG + 7 time-point of MS (Figure 3B), and positively correlated with the expression profiles of miR-449c.1 and the other members of the family.

## 4. Discussion

The endometrium undergoes periodic changes throughout a woman’s reproductive life, with each cycle involving proliferation, differentiation, breakdown, and regeneration. Our study linked the miR-449 family to the receptive phase of the hCG-primed endometrial cycle in healthy women. We extended the data from the previous sRNA-seq-based analysis by identifying a larger number of DE members of miR-449a/c, including template and non-template isomiRs (probably due to greater sequencing depth and the low heterogeneity of endometrial samples). For the first time, the expression profile of this miRNA family was followed through the successive phases of the endometrial cycle by RT-qPCR, and a peak of accumulation in the RE, followed by a decline, was verified.

Accumulating evidence suggests crosstalk between NOTCH and WNT/β-catenin signaling during endometrial remodeling. In the human endometrium, Notch family members were found to be expressed across the menstrual cycle [49]. Notch signaling has been implicated in endometrial remodeling events such as multiciliogenesis [50] and decidualization [49], in the surface epithelial cells and stromal cells, respectively. At the early stages of multiciliogenesis, suppression of the Notch signaling pathway by members of the miR-34/449 family [38], and the resulting cell-cycle exit [36], enhances the commitment of dividing progenitor cells to the fate of multiciliated cells in different systems of vertebrates. Subsequently, the miR-34/449 family is necessary for establishing and maintaining this fate via the regulation of genes involved in centriole amplification [51] and cilia formation [52]. During decidualization, endometrial stromal fibroblasts transform into specialized secretory decidual cells in the mid-secretory phase of the menstrual cycle, regardless of conception/pregnancy [49,53]. In the preimplantation uterus of mice, Notch1 mediates uterine stromal-differentiation and promotes decidualization [54]. A recent study demonstrated the presence of primary cilia in the decidual stromal cells of pregnant women. Furthermore, they observed that the number of ciliated decidual stromal cells in recurrent miscarriage is significantly lower than in the control group, indicating that stromal cell ciliogenesis is essential for normal pregnancy [55].

Recently, spatial reference maps of the human uterus and three-dimensional endometrial organoid cultures were generated [56]. Based on these, mapping the temporal and spatial dynamics of the human endometrium demonstrated the opposing roles of WNT and NOTCH signaling in cell fate-specification. WNT dominates in the early-secretory phase to maintain the ciliated lineages, while NOTCH is predominant in the mid- and late-secretory phases, to promote efficient differentiation toward the secretory lineages. Our study showed dynamic changes in the expression of miR-449a/c family members during the secretory phase, which peaked at hCG + 7, followed by a decline at hCG + 9. We can hypothesize that the miR-449 family may help to resolve the competition between the two signaling pathways, by initially exerting a negative control on NOTCH signaling in order to maintain the ciliated lineage, which is then attenuated to allow NOTCH activation and secretory lineage commitment.

While during a woman’s reproductive life the endometrium undergoes complex regular cycles, throughout perimenopause, ovarian activity decreases and affects the endometrium [57]. Initially, ovulation is sometimes unsuccessful, no corpus luteum is formed, and no progesterone is secreted from the ovary. Premenopausal menstrual cycles are therefore shortened and often irregular, due to anovulation or improper maturation of follicles [58,59]. In our case study, we analyzed the expression profiles of miR-449c.1 (the most highly represented and altered member of the family) separately in two women, one of whom was of reproductive age (a 34-year-old, with regular cycle phases, according to histological evaluation) and the other in the perimenopause (a 49-year-old, with shortened secretory phase, according to histological assessment). Unlike the woman of reproductive age, no increase in the levels of miR-449c.1 and its precursor in hCG + 7 was found in the woman in the perimenopause. This observation is further evidence of the role of the miR-449 family in controlling endometrial receptivity at a molecular level. The positive correlation between the levels of miR-449c and its isoforms, on the one hand, and their precursor, on the other hand, suggests that the control of their expression most likely occurs at the transcriptional level, without, of course, excluding the possibility of controlling the primary transcript and precursor processing. Recently, functional dysregulations of the ciliary process have been linked to maternal age [60]. Our case-study findings point to a relationship between the miR-449 family and the genetic regulation of endometrial aging. This association may be mediated through the ciliated lineage of endometrial epithelial cells [56], and/or ciliogenesis in decidual stromal cells [55].

This study has some limitations. The number of patients was not large, but this is common for assays requiring invasive sampling. Endometrial biopsies were taken from the same individuals at four time-points of the same menstrual cycle, potentially affecting gene expression at each subsequent time-point, due to local damage caused by the previous biopsy.

## 5. Conclusions

In our study, we observed that miR-449a/c and associated isomiRs could together be linked to human endometrial receptivity. Recently, miR-449a was shown to have an important impact on caprine endometrial-stromal-cell apoptosis and mice endometrial receptivity [61]. The miR-449 family is well known for helping to ensure proper cellular function and tumor suppression by mediating specific signaling pathways [38,43]. All this explains the great efforts aimed at elucidating the regulatory role of this miR family in healthy and diseased individuals, in particular with regard to reproductive functions and related disorders.

## Data Availability

Data are contained within the article or Supplementary Material.

## Supplementary Materials

The following supporting information can be downloaded at: https://www.mdpi.com/article/10.3390/biology12010055/s1: Table S1: list of SL primers, TT SL primers, TaqMan probes for miRs, isomiRs, and pre-miRs; Table S2: DE miR and isomiRs in hCG + 9 vs. proliferative phase; Figure S1: differential expression of miRs and isomiRs–MS phase compared with proliferative phase (Limma, Galaxy Version 3.38.3).
