# Supplementary figures and images for: The Dynamics of miR-449a/c Expression during Uterine Cycles Are Associated with Endometrial Development
Source: Biology (Basel). 2022 Dec 29;12(1):55. doi: 10.3390/biology12010055 (PMC9855972; doi:10.3390/biology12010055)

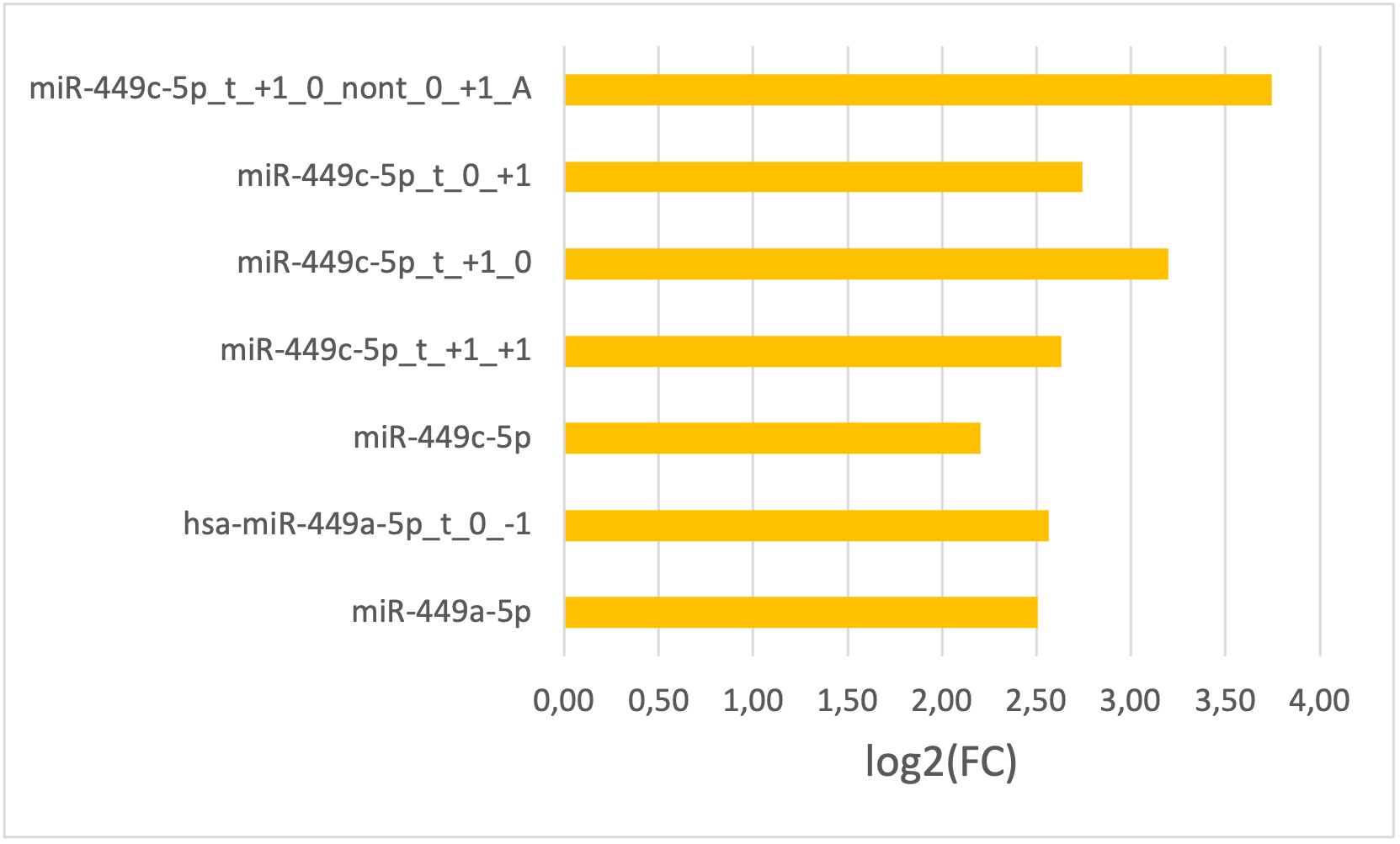

Supplement: Supplementary file 1 [file biology-12-00055-s001.zip › Figure S1.jpg]
