# Supplementary material for: The Dynamics of miR-449a/c Expression during Uterine Cycles Are Associated with Endometrial Development
Source: Biology (Basel). 2022 Dec 29;12(1):55. doi: 10.3390/biology12010055 (PMC9855972; doi:10.3390/biology12010055)
Supplement: Supplementary file 1 [file biology-12-00055-s001.zip › Table S1 _RT-qPCR_Primers and probes.doc]

*Table S1*. List of SL primers, TT SL primers, TaqMan probes for miRs, isomiRs, and pre-miRs

| Pre-miR-449c | 5’-3’ |
| --- | --- |
| 449c precursors -FW | GCTGGGATGTGTCAGGTAGG |
| 449c-precursors-R | TGAATGCAACAGAGAGGAGTG |
| RNU48 –FW | GATGACCCCAGGTAACTCTG |
| RNU48_R | AGAGCGCTGCGGTGATGGC |
|  |  |
| miR-449a-5p mature |  |
| Taq 449a | GATACGACACCAGCTA |
| SL 449a-5p | GTCGTATCCAGTGCAGGGTCCGAGGTATTCGCACTGGATACGACACCAGC |
| 449a -FW | GGCCGTGGCAGTGTATTGTTA |
| 449a -R- (UR) | GTGCAGGGTCCGAGGT |
|  |  |
| miR-449c-5p mature |  |
| Taq 449c | GATACGACACAGCCGC |
| SL 449c-5p | GTCGTATCCAGTGCAGGGTCCGAGGTATTCGCACTGGATACGACACAGCC |
| 449c-FW | GCCAGGCAGTGTATTGCTAGC |
| 449c -R- (UR) | GTGCAGGGTCCGAGGT |
|  |  |
| RNU48 |  |
| Taq RNU48- | GATACGACGGTCAGAG |
| SL RNU48- | GTCGTATCCAGTGCAGGGTCCGAGGTATTCGCACTGGATACGACGGTCAG |
| RNU48-FW | CCAGGTAACTCTGAGTGTGTC |
| RNU48-R- (UR) | GTGCAGGGTCCGAGGT |
|  |  |
| miR-449c.1-5p |  |
| Taq 449c.1 | GATACGACCAGCCGCT |
| SL 449c.1-5p | GTCGTATCCAGTGCAGGGTCCGAGGTATTCGCACTGGATACGACCAGCCG |
| 449c.1-FW | GCGCAGGCAGTGTATTGCTAG |
| 449c.1-R- (UR) | GTGCAGGGTCCGAGGT |
|  |  |
| miR-449c-5p mature TTRT |  |
| Taq 449c | GATACGACACAGCCGC |
| SL 449c-5p- TTRT | ACTGCCTAACATGCTCGCCAGGTACAGTTGGTACCTGTGATACGACACAGCC |
| 449c-FW- TTRT | GCCTAGGCAGTGTATTGCTAGC |
| 449c-R- TTRT | ACTGCCTAACATGCTCGCCAG |
|  |  |
| miR-449c.1-5p TTRT |  |
| Taq 449c.1- TTRT | GATACGACCAGCCGCT |
| SL 449c.1-5p- TTRT | CACTGCCTACATGCTCGCCAGGTACAGTTGGTACCTGTGATACGACCAGCCG |
| 449c.1-FW TTRT | GCGCAGGCAGTGTATTGCTAG |
| 449c.1-R- TTRT | CACTGCCTACATGCTCGCCA |
|  |  |
| RNU48-TTSL |  |
| Taq RNU48- TTRT | GATACGACGGTCAGAG |
| SL RNU48- TTRT | TCATCACTACATGCTCTCCAGGTACAGTTGGTACCTGTCTCCACTTGGTCAG |
| RNU48-FW TTRT | CCAGGTAACTCTGAGTGTGTC |
| RNU48-R- TTRT | TCATCACTACATGCTCTCCAG |
